# Vegetable Size Measurement Based on Stereo Camera and Keypoints Detection

**DOI:** 10.3390/s22041617

**Published:** 2022-02-18

**Authors:** Bowen Zheng, Guiling Sun, Zhaonan Meng, Ruili Nan

**Affiliations:** College of Electronic Information and Optical Engineering, Nankai University, Tianjin 300350, China; zhengbwen@mail.nankai.edu.cn (B.Z.); mengzn@mail.nankai.edu.cn (Z.M.); nrl@mail.nankai.edu.cn (R.N.)

**Keywords:** vegetable size measurement, computer vision, stereo camera, keypoints detection

## Abstract

This work focuses on the problem of non-contact measurement for vegetables in agricultural automation. The application of computer vision in assisted agricultural production significantly improves work efficiency due to the rapid development of information technology and artificial intelligence. Based on object detection and stereo cameras, this paper proposes an intelligent method for vegetable recognition and size estimation. The method obtains colorful images and depth maps with a binocular stereo camera. Then detection networks classify four kinds of common vegetables (cucumber, eggplant, tomato and pepper) and locate six points for each object. Finally, the size of vegetables is calculated using the pixel position and depth of keypoints. Experimental results show that the proposed method can classify four kinds of common vegetables within 60 cm and accurately estimate their diameter and length. The work provides an innovative idea for solving the vegetable’s non-contact measurement problems and can promote the application of computer vision in agricultural automation.

## 1. Introduction

Agricultural automation significantly improves the efficiency of agricultural production through IntelliSense and automation technology. In recent years, computer vision (CV) and deep learning have been widely applied in agricultural production processes (e.g., plant disease recognition, weed detection, yield prediction and non-contact size estimation). Producers can obtain many parameters and information about the crops through images with the help of CV. Compared with manual measurement, automatic measurement is a method with unified standards, high efficiency, and good real-time performance. Therefore, non-contact crop measurement based on CV and deep learning is an important research direction.

Early vision-based detection methods mainly utilized surface features such as color and lines to simply describe the crops’ quality [1,2]. They had not fully exploited the deeper-level information hidden in the images. It has become easier to learn the image structural features due to the development of deep learning. Researchers gradually tend to detect the crops’ quality using learning-based method. Dang [3] proposed a fruit size detection method based on image processing, which can estimate the diameter of circular fruit using natural images. Vivek [4] proposed a method to estimate the volume and mass of axi-symmetric fruits based on image processing. Sa [5] established a fruit detection system named DeepFruits, which combines the detection results of color and near-infrared images to achieve good results. In [6], Rabby proposed a fruit classification and measurement method based on edge detection, which can briefly describe the color and size of fruits. A mango size estimation method using Red-Green-Blue (RGB) image and depth camera was proposed in [7], which used the bounding box to measure the size of mangoes. Phate [8] developed a computer vision method to measure the sweet lime using dimensional analysis and normal regression. Sobol [9] used CIE L*a*b* method to evaluate the color coefficients of fried potato products. In [10], Ashtiani used multi-linear regression and neural networks to measure the size of almonds.

Existing vegetable and fruit size measurement methods are mainly based on simple image processing, such as edge detection [3,6] and bounding box [7]. Though these methods work sufficiently on regularly shaped fruits, working on complex shapes is not easy. In addition, these methods usually require the distance between the camera and the fruit as a priori information. They are only suitable for the fruits classification after picking. Some works introduce the depth value to complete the size measurement of the fruit on the tree [7]. However, these methods only work on a single type of fruit [7,8,10].

Keypoints detection was originally proposed for human pose estimation [11]. Some recent work has proved that it has a good effect in agricultural automation. Rong [12] proposed a tomato peduncle detection method for autonomous harvesting based on an improved you only look once (YOLO) method and RGB-D camera. Sun [13] proposed a multi-level feature fusion method for citrus bearing branch keypoint detection. In [14], Weyler proposed a leaf keypoint detection method to estimate the count of sugar beet leaves. Gan [15] developed a method to detect social behaviors among preweaning piglets using keypoint-based spatial and temporal features. Suo [16] proposed a stereo keypoint detection method to estimate the fish posture and length.

Keypoint detection is usually used to locate the stalk in automatic picking [12,13], guiding the robot to harvest fruits automatically. However, post-processing is still necessary to estimate the fruits’ size. Similar to human pose estimation, keypoint detection is usually used to recognize the animals’ postures and behaviors automatically in aquaculture [15,16]. In [16], Suo combined deep neural networks and binocular cameras to locate 3D coordinates of fishes’ keypoints, and finally estimated their length successfully. However, vegetable size estimation is another problem. Due to self-occlusion problems, binocular cameras will lose the depth of the object’s edge when measuring vegetables, and it worsens as the distance decreases. Therefore, it is difficult to locate the 3D position of keypoints on the edge of vegetables. We avoid the problem of depth loss by projecting the pixel distance of keypoints on RGB images into 3D space.

In summary, this paper proposes a size measurement method for gourd and solanaceous vegetables based on stereo camera and keypoints detection. This method combines the binocular stereo camera and the keypoints detection method to solve the non-contact vegetable size measurement problem without distance prior. In this work, we labeled a standard common objects in context (COCO) data set with 1600 images to train keypoints detection networks. To estimate the vegetable’s size, the method firstly obtains the RGB image and depth map of the object using a RealSense depth camera. Then, the pre-trained keypoint region-based convolutional neural networks (RCNN) classifies vegetables and locates keypoints at multiple scales. According to the keypoints’ location in the RGB image and the values in the depth map, we can calculate the distance between keypoints using the fusion method proposed in this paper. Finally, the method estimates of vegetables’ size according to the distance between keypoints. The method is suitable for automatic picking and classification for the advantages of simple deployment, high accuracy and low cost. We believe that this work will promote the application of machine vision and deep learning in agricultural automation. The main contributions of this paper are as follows:

- This paper proposed a non-contact vegetable size measurement method based on keypoint detection and a stereo camera. The method adopts a stereo camera to obtain RGB images and depth maps, then locates six keypoints using the object detection platform named Detectron2. To estimate the vegetables’ size, we propose a method to calculate the diameter and length of objects by fusing the pixel coordinates and depth values of keypoints.
- This paper proposes a vegetable keypoints location method based on depth cameras and object detection. To improve the performance of object detection and keypoint location, we designed a multi-scale strategy named zoom-in. The proposed methods can accurately classify the vegetables and locate six keypoints. The method can not only be applied to the vegetable size estimation but also be used to guide the manipulator to pick and classify vegetables automatically.
- This paper labeled and published a vegetable keypoints data set in standard COCO format with 1600 pictures, including four common vegetables: cucumber, eggplant, tomato, and pepper. Each target contains a region of interest (ROI) and six keypoints. This data set can be widely applied, ranging from classification, 3D position, size detection, manipulator picking, and many other fields.

This study is organized as follows. In Section 1, the background of the vegetable size measurement is briefed, the significance, contributions and the fundamental idea of the proposed method are summarized. Section 2 elaborates the structure and procedure of the vegetable size detection method and introduces the keypoints detection data set of vegetables labeled in this work. Section 3 gives results of the vegetable size detection experiments. Section 4 discusses the effectiveness of our method based on the results. Section 5 summarizes the article and puts forward the prospects for subsequent works.

## 2. Materials and Methods

**Figure 1:**
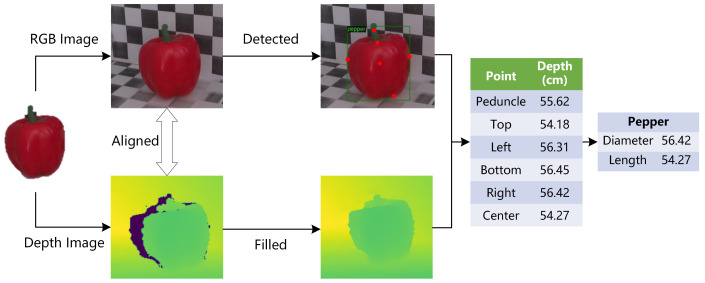
The overall structure of vegetable size measurement method.

Aiming to provide a method of non-contact measurement for vegetables in agricultural automation, this paper proposes a novel method for vegetable size measurement at adaptive distance. Based on the RealSense depth camera and the Detectron2 object detection platform, this method can complete the process of image acquisition, recognition and size measurement without the prior of distance between the target and the camera. Figure 1 shows the overall structure of the method proposed in this paper. RealSense camera takes and aligns the target’s RGB image and depth map. In the RGB image processing process, the Detectron2 platform is used to identify the vegetable’s type and locate six key points(i.e., Peduncle, Top, Left, Bottom, Right and Center). Meanwhile, the original depth map with holes is filled by multiple filters in the depth map processing. Then, we obtain the depth value of key points by querying corresponding points in the depth map. Finally, we can get the object’s size by fusing the pixel coordinates and depth of the keypoints. The method can be divided into four modules: color image and depth map acquisition, multi-scale target detection, keypoints detection and size estimation.

### 2.1. Color Image and Depth Map Acquisition

We use RealSense D415 camera to sense the RGB image and depth map of the target vegetable. The size of the RealSense D415 camera is 99 mm × 20 mm × 23 mm, weighing about 75 g, using active IR stereo depth technology. The view field angle of the depth camera is $65^{\circ }\times \;40^{\circ }$. The frame rate can reach 90 fps at the maximum resolution resolution of 1280×720. The view field angle of RGB camera is $69^{\circ }\times 42^{\circ }$. The frame rate can reach 30 fps at the maximum resolution resolution of 1280×720. The focal length of RGB camera is 1.88 mm, and the size of image sensor is 2.73 mm ×1.55 mm. RealSense D415 can effectively measure the depth of the target in the range of 0.3 m–10 m.

**Figure 2:**
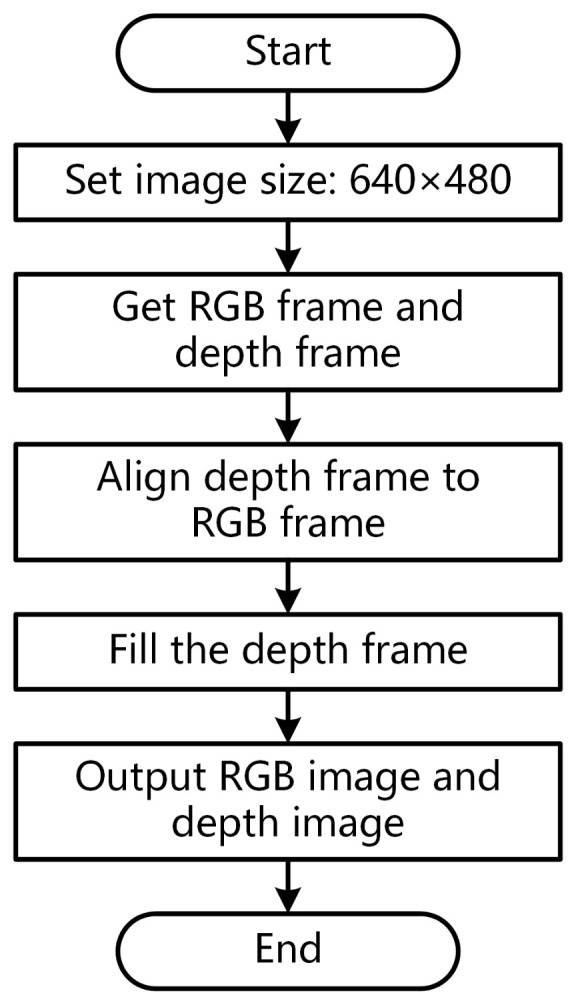
The process of acquiring RGB image and depth map.

Figure 2 shows the process of acquiring and processing RGB image and depth map. First, we set the resolution of color image and depth map to 640×480. We do not use the maximum resolution supported by RealSense D415 because a low resolution provides a lower depth detection limit. The minimum depth is 310 mm when the resolution is set to 640×480.

We acquired the RGB frame and depth frame after setting the resolution. However, the depth map and RGB image used different reference systems because of the different positions of cameras. That leads to the inconsistency of the detection target’s position in the depth map and the color image. We have to align them before further processing. The basic principle is converting the 2D points in depth maps to 3D space first and then projecting the points in 3D space to the plane of RGB cameras. Finally, we can query the depth value of each point in the RGB image at the corresponding position in the depth map.

The depth camera used the left camera as the matching reference to calculate the depth. Therefore, it lost the depth of points contained in the left camera but not in the right camera. It returned 0 when we query the depth of keypoints on the vegetable’s left edge. Thus, we used two filters in the pyrealsense2 toolkit, spatial_filter and hole_filling, to repair the depth map.

Finally, the image acquisition module will output the vegetable’s RGB image and depth map for subsequent modules.

### 2.2. Keypoint Detection Networks

**Figure 3:**
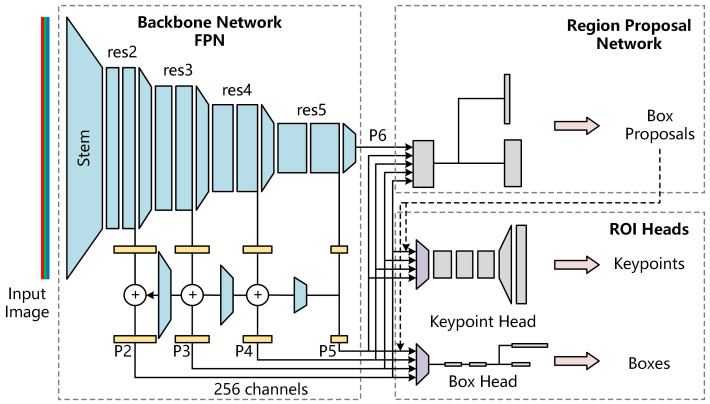
The structure of keypoint detection networks.

We recognized the vegetable and locate the keypoints using the keypoint RCNN provided by Detectron2. Figure 3 shows the structure of keypoint detection networks. It extracted the feature maps of the input image at different scales using feature pyramid networks (FPN). Firstly, the backbone network used a ResNet named Stem to preliminarily extract the features of three-channel RGB images and output 64 channel feature maps. Then, it used four ResNets (i.e., res2, res3, res4, and res5) to extract features at different scales in turn. The features output, finally, using FPN, were P2 (1/4 scale), P3 (1/8 scale), P4 (1/16 scale), P5 (1/32 scale) and p6 (1/64 scale). All feature maps contain 256 channels.

With the feature maps at five different scales as input, the regional proposal network (RPN) detected the object regions and calculated their objectness and anchor box. The objectness is the probability that a region contains an object, and the anchor box indicates the region’s position on the original image. RPN finally provides 1000 proposal boxes with the highest objectness at the ROI heads.

ROI heads used in this paper include keypoint head and box head. With the feature maps and proposal boxes as input, the keypoint head used ROIAlign (the purple trapezoid in Figure 3) to obtain a fixed feature map with a size of 14×14 first. Then the keypoint heat map was calculated using a multi-layer convolution and one-layer deconvolution. Finally, it outputs the coordinates of the keypoints according to the key point heat map. Box head, firstly, obtained a fixed feature map with a size of 7 × 7 using ROIAlign. Then it expanded the feature map to a one-dimensional vector, and finally outputted the position and classification of each box through multiple fully connected layers.

It will discard all proposal boxes with classification scores less than 0.6 in this paper and output the boxes and keypoints of all vegetables contained in the image.

### 2.3. Multi-Scale Object Detection

Theoretically, the keypoint detection network can detect small objects in the image because it extracts image features at multiple scales. However, in experiments, we found that if the image contains an extensive range of background areas, it is usually difficult to predict the small objects’ classification and keypoints accurately. It cannot get a high classification value generally for small objects when the training set is not large enough because the detection networks only use low-level feature maps.

**Figure 4:**
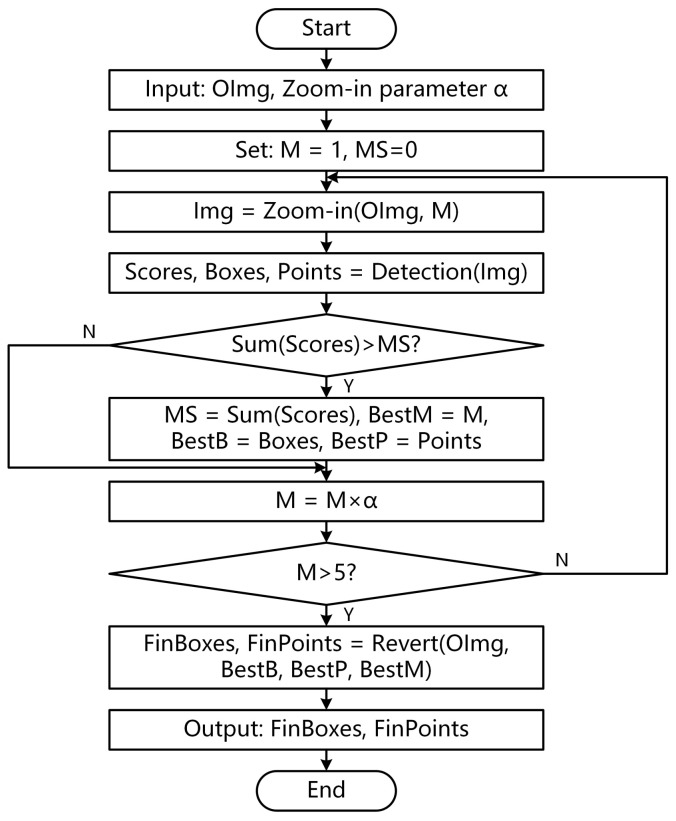
The flow chart of multi-scale scale object detection.

To solve this problem, this paper proposes a multi-scale detection module named zoom-in. Figure 4 shows the implementation details of the module.

1. Input the image to be tested and zoom-in parameter α.
2. Set the magnification to M=1 and the maximum score to MS=0.
3. Execute the detection loop. Magnify the original image to *M* times using the zoom-in function and obtain a new image named Img. The execution detail of zoom-in is to magnify the original image to M times with Bilinear Interpolation, then cut a new image with the same size as the original image in the center of the magnified image.
4. Calculate the score of Img using the pre-trained key point detection networks. Furthermore, detect the box and keypoints contained in the image.
5. Sum the scores of all proposal boxes. If the total score is greater than MS, update the maximum score *MS* and record the best magnification bestM as *M*. Otherwise, go to the next step.
6. Update magnification to M=M×α. If M>5, the module will jump out of the loop. Otherwise, it will return to 3.
7. Map the boxes and keypoints on Img back to the original image with Revert according to the magnification and output FinBoxes and FinPoints. The execution detail of the Revert is to calculate the center point of the image according to the size of the original image, then shorten the distance between the predicted points and the center point by BestM times.

### 2.4. Vegetable Size Estimation

Keypoint detection networks output the pixel coordinates (x,y) of the keypoints on the RGB image. The depth map provides the distance *d* between the keypoint and the camera. There are two ways to calculate the distance in 3D space according to the mentioned information:

• Calculate the 3D space coordinates of keypoints by projecting them to the 3D space. Then, use the 3D coordinates to calculate the distance between the two points directly.
• Calculate the pixel distance d between two points in the RGB image, then map the pixel distance to the 3D space. Meanwhile, correct the distance error caused by the depth difference between different key points with mathematical methods.

Theoretically, the first method gives a more accurate distance. However, in practical applications, we found that binocular depth cameras usually lose the depth of keypoints at the edge of the object. On the contrary, it is easy to obtain the object’s center point’s depth. Meanwhile, the diameter and length of the target vegetable are much less than the depth. Thus, the second method calculates the distance between keypoints more accurately.

**Figure 5:**
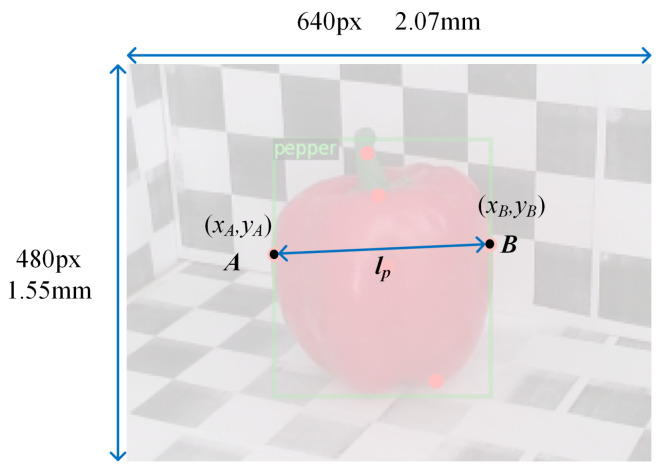
Pixel distance between two points in image.

In Figure 5, $A(x_{A},y_{A})$ and $B(x_{B},y_{B})$ are two keypoints in RGB image. The pixel distance between the two points is:

(1) $$l_{p}=\sqrt{{\left(x_{A}-x_{B}\right)}^{2}+{\left(y_{A}-y_{B}\right)}^{2}}.$$

The corresponding size of the image with a resolution of 640×480 on the actual image sensor is 2.07×1.55 mm. Therefore, the actual distance between two points on the image sensor is:

(2) $$l_{s}=\frac{1.55l_{p}}{480}\mathrm{mm}.$$

**Figure 6:**
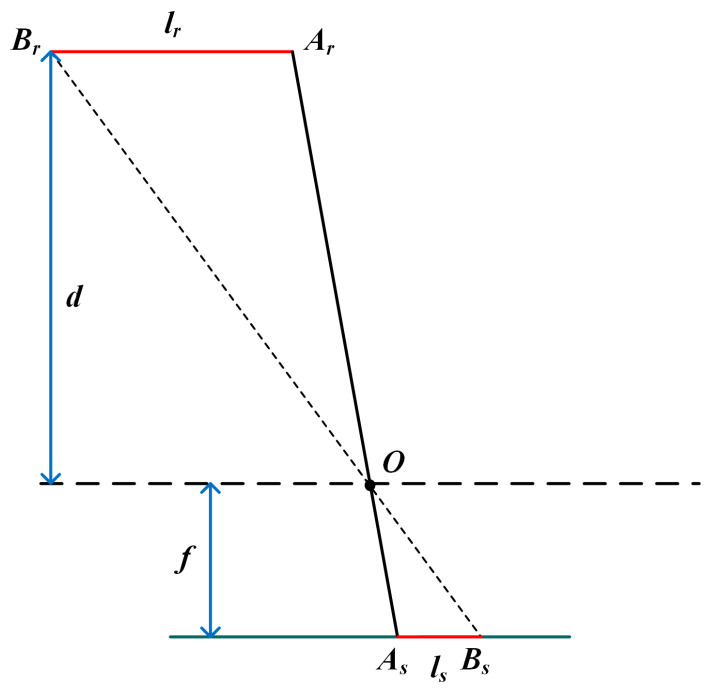
The principle of calculating distance between two keypoints.

Figure 6 shows the principle of calculating the actual distance between two points in 3D space according to the length on the image sensor. *O* is the optical center of the RGB camera. $A_{s}$, $B_{s}$ is the projection of two keypoints on the image sensor. *f* is the focal length of the RGB camera, and *d* is the object’s depth. According to the similarity of $▵OA_{r}B_{r}$ and $▵OA_{s}B_{s}$, the actual distance is:

(3) $$l_{r}=\frac{l_{s}d}{f}.$$

Vegetables are 3D objects with thickness. Generally, the edge of the object has a greater depth than the center. Therefore, we have to correct depth error while estimating the diameter and length of vegetables. Fortunately, the cross-section of gourd and solanaceous vegetables is usually circular. Thus, the depth difference between the central and edge points is equal to the radius. In this paper, we take the distance between left and right keypoints as the diameter *D* of the vegetable. It is assumed that the distance between the projection of the left and right key points on the image sensor is $D_{s}$, and *d* is the depth of the center point. We have:

(4) $$\frac{D}{D_{s}}=\frac{d+D/2}{f}.$$

Thus,

(5) $$D=\frac{dD_{s}}{f-D_{s}/2}.$$

We take the distance between the top and bottom key points as the length *L* of vegetables, and the distance between top and bottom keypoints projected on the image sensor is $L_{s}$. We have:

(6) $$\frac{L}{L_{s}}=\frac{d+D/2}{f}.$$

Thus,

(7) $$L=\frac{dL_{s}}{f-D_{s}/2}.$$

### 2.5. Datasets

**Figure 7:**
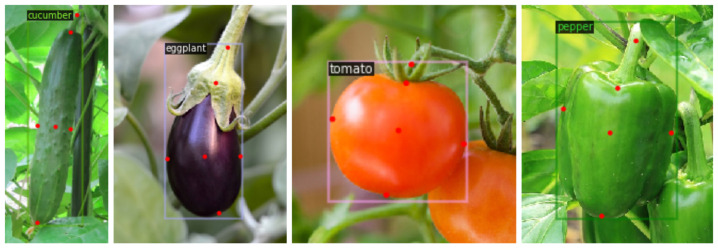
Some samples of the data set.

To train the keypoint detection networks, we collected and labeled the vegetable keypoint data set. The data set contained 1600 images, including four common vegetables: cucumber, eggplant, tomato and pepper. Each category contains 400 pictures, of which 320 are training sets and 80 are test sets. Interested readers can obtain this data set from the following link: https://github.com/BourneZ130/VegetableDetection/tree/main/Dataset, accessed on 15 February 2022. Figure 7 shows some samples. The main object in each image includes a ROI box and six keypoints: Peduncle, Top, Left, Bottom, Right and Center. Peduncle is a point on the vegetable’s handle. Top is the point closest to the peduncle on the vegetable. Bottom is the point farthest from the peduncle. Left and Right are the left and right endpoints of the widest segment perpendicular to the central axis on the vegetable. Center is the vegetable’s visual center point. We label the ROI box and keypoints using a software tool named Labelme, then convert the data set annotation file to standard COCO format. COCO is a data set provided by the Microsoft team for image detectrion. Labelme is obtained from the following link: https://github.com/wkentaro/labelme, accessed on 7 January 2022.

This work adopted six keypoints to estimate the vegetable’s size, because fewer keypoints are helpful to simplify the size estimation model and make it easier to label the image sets, which makes the method easier to apply. In addition, the results in [16] prove that estimating the object’s size with the distance between two keypoints offers better accuracy than measuring the curve fitted by more keypoints. Adopting six keypoints is appropriate for the four types of vegetables in our work. The Left and Right keypoints are used to estimate the diameter of vegetables, the Top and Bottom keypoints are used to measure the length, and the Center keypoint is used to determine the object’s depth. Peduncle keypoint is the picking point reserved for automatic harvesting.

## 3. Results

### 3.1. Experimental Settings

**Figure 8:**
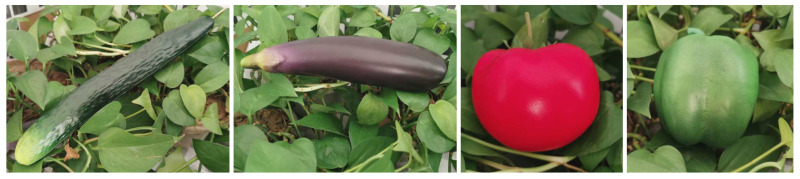
Vegetable models used to evaluate the methods.

We evaluated the size measurement effect of the proposed method with some model vegetables. Figure 8 shows some models we used in the experimental scenario. Three different models were used for each vegetable. Table 1 shows the standard parameters of each model. The experimental scene was a simulated farmland environment with green leaves as background built in the laboratory. The lighting condition is uniform natural sunlight, and the illumination intensity is about 3000lx.

All the experiments are implemented on a workstation with Intel(R) Xeon(R) W-2145 @3.70 GHz CPU, 64.0GB DDR4 memory, and NVIDIA Quadro RTX4000 GPU. The keypoint detection networks are trained with a learning rate of 0.0025, maximum iteration of 30,000 and a batch size of 16. The implementation code of our size estimation method can be accessed on https://github.com/BourneZ130/VegetableDetection, accessed on 15 February 2022.

This paper used the correct rate to evaluate the classification performance. It is calculated using Equation (8).

(8) $$CR=\frac{T_{r}}{T_{n}}\times 100\%,$$

where $T_{n}$ is the total experimental number, and $T_{r}$ is the number of successful recognitions.

This paper use the mean absolute percentage error (*MAPE*) to evaluate the size measurement performance. $MAPE_{D}$ denotes the results of diameter and $MAPE_{L}$ is the results of length. The *MAPE* is calculated with Equation (9).

(9) $$MAPE=\frac{1}{T_{n}}\sum_{i=1}^{T_{n}}\frac{\left|X_{pi}-X_{ai}\right|}{X_{ai}}\times 100\%,$$

where $X_{pi}$ is the predicted distance and $X_{ai}$ is the actual value in *i*th test.

### 3.2. Results of Vegetables Classification

This section evaluates the method’s accuracy in classifying four types of vegetables at different depths. In the experiments, we used the RealSense D415 to obtain RGB images of vegetables at different distances. The resolution of the image is set to 640×480. We employed the zoom-in strategy described in Section 2.3. The details show the image’s center view field is zoomed in 1.2× in each iteration. The test depth is 40–160 cm with an interval of 20 cm. We used three samples for each vegetable, and performed 100 measurements at different angles for each sample at the same depth. In the experiments, we marked a successful detection if the predicted class was the same as the ground truth. If the predicted class was different from the ground truth, or no vegetable was detected, was classed as a failed detection.

**Figure 9:**
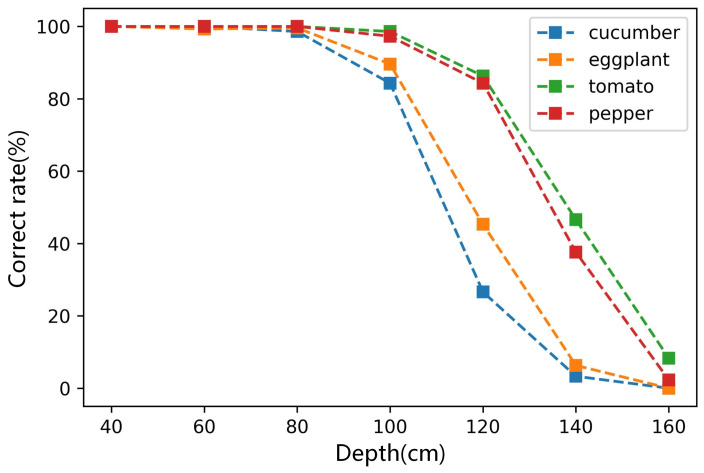
The correct rate of classification at different depths.

Figure 9 shows the correct rate of classification for four types of vegetables at different depths. It is shown that when the depth is less than 80 cm, the correct rate for the four vegetables is very high, close to 100%. When the depth is greater than 80 cm, the success rate decreases gradually with the depth increase. That of cucumber and eggplant decreased faster. When the distance reach 160 cm, the correct rate was almost 0. That of tomato and pepper decreased slower, and the correct rates were almost 100% when the depth was 100 cm. Therefore, the method’s correct rate for round vegetables is higher than in long strip vegetables.

### 3.3. Results of Size Estimation

This section evaluates the method’s performance of size estimation for four types of vegetables at different depths. In experiments, we used RealSense D415 to obtain RGB images and depth maps of vegetables at different depths. The resolution of the image is set to 640×480. The zoom-in strategy with α=1.2 was applied. The test depths are 40 cm, 60 cm, 80 cm and 100 cm, respectively. Three samples were used for each vegetable, and each sample was measured 100 times at different angles at the same depth.

Table 2 shows the MAPE of diameter and length estimation for 12 vegetable samples at different depths. It is shown that the MAPE for different samples of the same vegetable was similar. For example, when the depth was 40 cm, the maximum MAPE of tomato diameter estimation was 2.02%, the minimum was 1.95%, and the difference was only 0.07%. The MAPE of tomato’s diameter was the smallest, which is only 2% at a depth of 40 cm, and still less than 8% when the depth reached 100 cm. The MAPE of cucumber’s diameter was the largest, which is greater than 6% at a depth of 40 cm. The MAPE of the eggplant’s length is the smallest, which is only 2% at a depth of 40 cm, and still less than 8% when the depth reaches 100 cm. The MAPE of the pepper’s length is the largest, which was about 7% at a depth of 40 cm.

**Figure 10:**
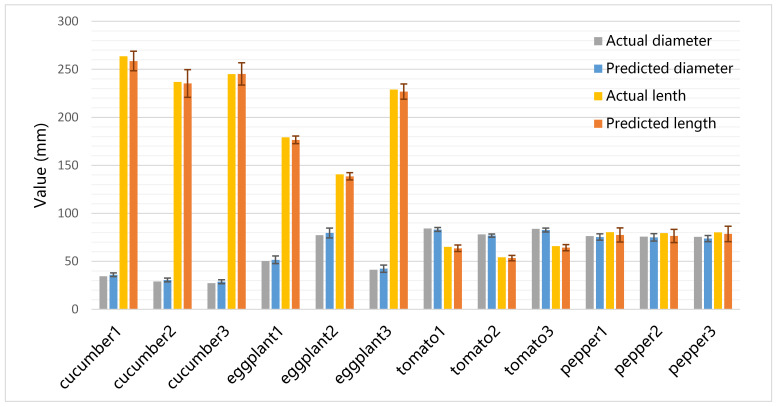
The actual and predicted size of vegetable samples at the depth of 60 cm. The error bars denote the standard deviation of 100 tests at different angles.

Figure 10 shows the predicted size of vegetable samples at a depth of 60 cm. It is shown that in 100 experiments, the average predicted size was very close to the actual size of vegetables, and the standard deviation is sufficiently small. The average predicted lengths of all vegetables were slightly smaller than the actual values. The average predicted diameters of cucumbers and eggplants were slightly greater than the actual values.

**Figure 11:**
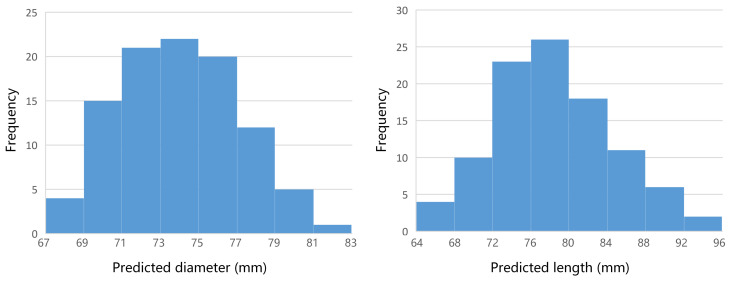
The distribution of predicted pepper size at the depth of 60 cm. Left is the result of diameter, and the actual value is 75.55 mm. Right is the length result, and the actual value is 80.17 mm.

Figure 11 shows the distribution of predicted pepper size at a depth of 60 cm. It is shown that in 100 experiments, the frequency of prediction diameters at (73 cm, 75 cm] is the highest, followed by (71 cm, 73 cm] and (75 cm, 77 cm]. The predicted diameters are generally normally distributed, and the mean value approached the actual value 75.55 cm. The frequency of prediction lengths at (76 cm, 80 cm] is the highest, followed by (72 cm, 76 cm]. They are also normally distributed, and the mean value is slightly smaller than the actual value 80, 17 cm. The distribution of the predicted size for the other three vegetables is similar to that of pepper.

### 3.4. Multiple Object Detection

This section evaluates the method’s performance for multiple object detection. In the experiments, the proposed method classified three objects of the same kind in the camera’s view and estimated their size at the same time. Table 3 shows the correct rate of classification and the MAPE of size estimation at different depths. The MAPE was slightly higher than that of a single object in Table 2, but the gap was minimal. Similar to the results in Figure 9, the proposed method can recognize four types of vegetables with a high probability at the 40 cm and 80 cm depths.

### 3.5. Comparison with State-of-Art Methods

This section compares the proposed method with size estimation methods based on edge detection and bounding box. The implementation details of edge-detection-based method refer to [3]. The implementation details of the bounding-box-based method refer to [7]. The resolution of the image is set to 640×480. The test depths were 40 cm, 60 cm, 80 cm and 100 cm, respectively. Three samples were used for each vegetable, and each sample was measured 100 times at different angles at the same depth.

Table 4 shows the MAPE of diameter and length estimation for three comparison methods at different depths. It is demonstrated that our approach performs better than two comparison methods at any depth. As these two comparison methods are mainly applicable to ellipsoidal vegetables, their performance on irregular vegetables (e.g., cucumber, eggplant and pepper) is terrible. The MAPE of tomato size estimation of our approach is also better than comparison methods. Additionally, the gap between the methods gradually increases as the depth increases. Therefore, the approach proposed in this paper is more effective. At the same time, compared to other methods, this paper proposes a more universal approach that can be applied to irregular vegetables.

## 4. Discussion

This work applies the keypoint detection model and depth camera on vegetable size estimation in an innovative way. Our experiments show that this method achieves good results. This section discusses the effectiveness of the proposed method based on results in Section 3.

### 4.1. Effectiveness of the Proposed Method

The non-contact vegetable size estimation method uses the keypoint RCNN in the Detctron2 platform to classify vegetables and locate six keypoints for each object. Then, the pixel coordinates of keypoints in the RGB image and the depth values in the depth map are fused to estimate the diameter and length of vegetables. Many works [17,18,19,20] indicate that small-object detection is a challenge for RCNN-based methods. Compared with regular-sized objects, small objects have less information as its proportion in the image is very small. Additionally, labeling the training data of small objects is difficult and requires high labor costs [21]. Thus, the accuracy of classification and keypoint location is greatly affected by proportion of vegetables in the image. Therefore, our method adopted a zoom-in module to solve the problem. This module works by increasing the proportion of objects in the view field.

Section 3.2 evaluates the classification performance at different depths. Figure 9 shows that when the depth is less than 80 cm, the classified correct rates for four types of vegetables are almost 100%, and they are still more than 80% when the depth reaches 100 cm. Although it is difficult to compare two methods on different data sets, when the depth is less than 80 cm, our method still performed better than methods in reviews [22,23]. Therefore, we believe that the proposed method can effectively classify four vegetables within 80 cm.

Section 3.3 evaluates the size estimation performance at different depths. Considering that the classification ability of this method decreases rapidly when the depth is greater than 100 cm, we evaluated the size estimation performance within 100 cm. As shown in Figure 10 and Table 2, our method has excellent accuracy in tomato size estimation. We also note that the size estimated error for tomato is slightly smaller than other three irregular vegetables. Bargoti’s [24] and Wan’s [25] studies also report similar results, namely, that RCNN-based methods perform better on regular objects (e.g., apple and orange) than irregular objects (e.g., mango and almond). How to improve the performance of RCNN-based methods on irregular objects is still an open question. Table 4 shows that our method performs better than compared methods based on edge detection and bounding box. Additionally, when the distance was less than 60 cm, the method controlled the MAPE for four types of vegetables within 8%. It is adaptable enough for vegetable size classification.

The results in Figure 9 and Table 2 show that the performance of the proposed method decreases with the increase in depth. The main reason is that RCNN-based methods are not very effective when used to detect small objects [18,19]. The object was not clear enough, even though the zoom-in strategy was used. The results in [26,27] indicate that the clarity and resolution of images influences the performance of RCNN-based methods. We may solve this problem by using a high-definition (HD) camera. At the same time, we note that a lot of work regarding image super-resolution technology has been published [28], which can expand image resolution while maintaining clarity. These technologies may help the zoom-in strategy to become more effective. In addition, deep neural networks are data-driven methods, and the quality of data sets significantly impacts keypoint detection. We can improve the method’s performance by providing larger training sets or adopting better annotation strategies.

In a real scenario, the proposed method usually needs to process multiple objects in the field of view simultaneously. Section 3.4 proves that the method also works for multiple objects detection. Another question is whether the vegetable we want to measure may be occluded by other vegetables. Point-cloud-based methods usually complete the whole object shape by deep learning models like PointNet [29] or dynamic graph convolutional neural networks (DGCNN) [30]. We plan to use invisible keypoint detection technology to solve this problem. Fortunately, the annotation of invisible keypoints in the occluded part is supported by the standard COCO format [31]. When estimating the size of vegetables, the diameter and length can be obtained by fusing the pixel distance of keypoints in the RGB image and the depth of visible keypoints. However, we have not completed this work because of the limitation of the current image set. In future work, we will collect and label more sufficient data sets to improve the performance of the vegetable size estimation method.

### 4.2. Effectiveness of Zoom-In Module

**Figure 12:**
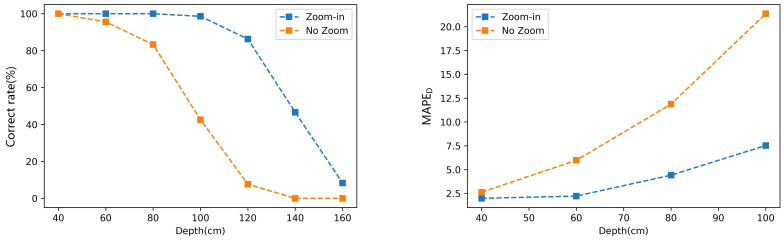
The method’s performance of classification and diameter estimation when the zoom-in module is used or not.

This paper proposes the zoom-in module to solve the problem of object detection when the distance increases. Before the classification and keypoint detection, the module first enlarges the central area of the view field by bilinear interpolation to obtain more accurate prediction results. After the prediction, the ROI boxes and keypoints are mapped back to the original images to estimate vegetables’ size. Figure 12 compares the method’s performance of classification and diameter estimation with zoom-in module or not. Figure 12 (left) shows the classification performance, and the correct rate is calculated by Equation (9). When the zoom-in module is used, the method can accurately classify the tomatoes within 100 cm. If the zoom-in module is not used, the classification performance decreases rapidly as the depth increases. The correct rate of classification is less than 50% when the depth is 100 cm. Figure 12 (right) shows the MAPE of tomato diameter estimation at different depths. It can be seen that the method’s performance with the zoom-in module is better than not at any depth. When the depth is small, the gap between them is not obvious. However, when the distance is 100 cm, the MAPE of the method without the zoom-in module is more than 20%, while that with the zoom-in module is only 7.5%. Therefore, the proposed zoom-in module can effectively improve the method’s performance of classification and size estimation.

## 5. Conclusions

This paper proposes a intelligent method for vegetable classification and size estimation based on object detection and depth camera. This method provides a feasible solution to the problem of non-contact vegetable measurement in the field of agricultural automation. In this work, we labeled a data set with 1600 images, containing four types of common vegetables. The method obtains the RGB image and depth map of the object using a RealSense D415 camera. Then, pre-trained keypoint detection networks process the RGB image to classify the vegetable and locate keypoints. Finally, we can estimate the vegetable’s size by fusing the keypoints’ pixel coordinates and depth. This method has broad application prospects in the field of automatic vegetable picking. It can promote the development and application of machine vision in the field of intelligent agriculture.

In future work, we will further improve the size estimation model for other vegetables. At the same time, we will try to use an HD camera and super-resolution reconstruction technology to improve the method’s performance at long distances. We can also combine a variety of depth cameras to improve the accuracy of keypoint location further.

## Figures and Tables

**Table 1:** The standard sizes of vegetable models.

Object	Diameter/mm	Length/mm	Object	Diameter/mm	Length/mm
Cucumber1	34.53	263.54	Tomato1	84.36	65.17
Cucumber2	29.03	236.96	Tomato2	78.10	54.28
Cucumber3	27.36	245.13	Tomato3	83.79	65.81
Eggplant1	50.28	179.22	Pepper1	76.41	80.34
Eggplant2	77.37	140.64	Pepper2	75.84	79.49
Eggplant3	41.31	229.05	Pepper3	75.55	80.17

**Table 2:** The MAPE of diameter and length estimation for 12 vegetables at different depths.

Object	40 cm		60 cm		80 cm		100 cm	
	${\mathrm{MAPE}}_{D}$	${\mathrm{MAPE}}_{L}$	${\mathrm{MAPE}}_{D}$	${\mathrm{MAPE}}_{L}$	${\mathrm{MAPE}}_{D}$	${\mathrm{MAPE}}_{L}$	${\mathrm{MAPE}}_{D}$	${\mathrm{MAPE}}_{L}$
Cucumber1	6.13%	3.21%	6.49%	3.64%	10.89%	7.23%	18.39%	13.90%
Cucumber2	7.24%	4.19%	7.93%	4.91%	11.23%	8.98%	19.77%	14.13%
Cucumber3	5.94%	3.28%	6.42%	3.96%	9.48%	7.69%	17.17%	14.47%
Average	6.44%	3.56%	6.95%	4.17%	10.53%	7.97%	18.44%	14.17%
Eggplant1	5.96%	1.96%	6.59%	2.18%	8.91%	4.03%	12.56%	7.17%
Eggplant2	5.27%	2.07%	5.89%	2.29%	9.58%	4.27%	13.09%	7.22%
Eggplant3	6.68%	2.43%	7.23%	2.89%	10.89%	4.94%	14.84%	7.89%
Average	5.97%	2.15%	6.57%	2.45%	9.79%	4.41%	13.50%	7.43%
Tomato1	1.99%	3.56%	2.15%	4.43%	4.25%	6.95%	7.37%	11.56%
Tomato2	2.02%	4.25%	2.28%	4.65%	4.68%	7.19%	7.99%	12.43%
Tomato3	1.95%	3.89%	2.24%	4.46%	4.33%	6.88%	7.22%	10.89%
Average	1.99%	3.90%	2.22%	4.51%	4.42%	7.01%	7.53%	11.63%
Pepper1	2.86%	6.56%	3.48%	7.98%	7.49%	11.64%	10.58%	14.56%
Pepper2	2.91%	7.25%	3.67%	7.86%	8.01%	11.48%	11.84%	14.83%
Pepper3	2.75%	6.89%	3.33%	8.13%	7.28%	11.99%	10.02%	14.21%
Average	2.84%	6.90%	3.49%	7.99%	7.59%	11.70%	10.81%	14.53%

**Table 3:** The results of multiple object detection at different depths.

Objects	40 cm			80 cm		
	${\mathrm{MAPE}}_{D}$	${\mathrm{MAPE}}_{L}$	CR	${\mathrm{MAPE}}_{D}$	${\mathrm{MAPE}}_{L}$	CR
Cucumber	6.89%	3.87%	100%	11.25%	8.53%	97.67%
Eggplant	6.34%	2.21%	100%	10.77%	5.03%	98.33%
Tomato	2.02%	3.96%	100%	4.93%	7.58%	99.33%
Pepper	2.95%	7.16%	100%	8.47%	12.69%	99.00%

**Table 4:** The vegetable size estimation results of three comparison methods at different depths.

Objects	Methods	40 cm		60 cm		80 cm		100 cm	
		${\mathrm{MAPE}}_{D}$	${\mathrm{MAPE}}_{L}$	${\mathrm{MAPE}}_{D}$	${\mathrm{MAPE}}_{L}$	${\mathrm{MAPE}}_{D}$	${\mathrm{MAPE}}_{L}$	${\mathrm{MAPE}}_{D}$	${\mathrm{MAPE}}_{L}$
Cucumber	Edge Detection	28.73%	6.18%	30.74%	7.89%	33.96%	12.24%	38.19%	19.18%
	Bounding Box	48.19%	24.84%	49.56%	26.87%	54.12%	29.10%	60.42%	34.93%
	Ours	6.44%	3.56%	6.95%	4.17%	10.53%	7.97%	18.44%	14.17%
Eggplant	Edge Detection	11.58%	4.12%	13.17%	4.87%	18.93%	7.98%	25.76%	12.89%
	Bounding Box	27.81%	10.65%	31.76%	14.98%	38.71%	18.12%	48.52%	20.70%
	Ours	5.97%	2.15%	6.57%	2.45%	9.79%	4.41%	13.50%	7.43%
Tomato	Edge Detection	2.21%	5.03%	3.31%	6.98%	7.11%	10.94%	13.25%	18.61%
	Bounding Box	2.54%	5.86%	4.17%	7.81%	7.29%	11.18%	14.13%	16.37%
	Ours	1.99%	3.90%	2.22%	4.51%	4.42%	7.01%	7.53%	11.63%
Pepper	Edge Detection	14.91%	13.37%	16.16%	15.52%	19.96%	19.48%	24.35%	25.67%
	Bounding Box	9.33%	8.52%	10.84%	10.03%	15.06%	14.23%	19.72%	18.63%
	Ours	2.84%	6.90%	3.49%	7.99%	7.59%	11.70%	10.81%	14.53%

## Data Availability

All data generated or analyzed during this study are included in this article.
